# Collisional energy transfer in the CO–CO system†

**DOI:** 10.1039/d2cp01065h

**Published:** 2022-04-25

**Authors:** Michał Żółtowski, Jérôme Loreau, François Lique

**Affiliations:** LOMC – UMR 6294, CNRS-Université du Havre 25 rue Philippe Lebon BP 1123 F-76063 Le Havre France francois.lique@univ-rennes1.fr; Univ Rennes, CNRS, IPR (Institut de Physique de Rennes) – UMR 6251 F-35000 Rennes France; KU Leuven, Department of Chemistry B-3001 Leuven Belgium

## Abstract

An accurate determination of the physical conditions in astrophysical environments relies on the modeling of molecular spectra. In such environments, densities can be so low (*n* ≪ 10^10^ cm^−3^) that local thermodynamical equilibrium conditions cannot be maintained. Hence, radiative and collisional properties of molecules are needed to correctly model molecular spectra. For comets at large heliocentric distances, the production of carbon monoxide (CO) gas is found to be larger than the production of water, so that molecular excitation will be induced by collisions with CO molecules. This paper presents new scattering calculations for the collisional energy transfer in CO–CO collisions. Using the quantum coupled states approach, cross sections and rate coefficients are provided between the first 37 rotational states of the CO–CO system. Cross sections were calculated for energies up to 800 cm^−1^, and excitation rate coefficients were derived for temperatures up to 100 K. In comparison with data available in the literature, significant differences were found, especially for the dominant transitions. Due to the high cost of the calculations, we also investigated the possibility of using an alternative statistical approach to extend our calculations both in terms of rotational states and temperatures considered. The use of these new collisional data should help in accurately deriving the physical conditions in CO-dominated comets.

## Abstract 2

This paper presents new quantum scattering calculations for the collisional energy transfer in CO–CO collisions. Cross sections and rate coefficients are provided between the first 37 states of the CO–CO system.

## Graphical abstract

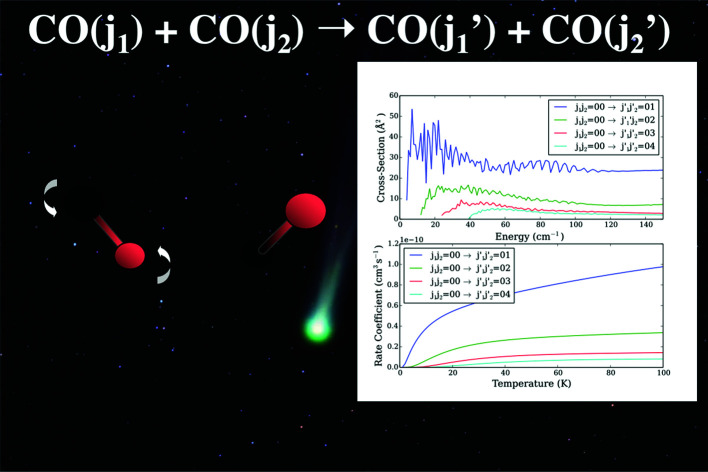

## 1 Introduction

Comets are valuable sources of information about the evolution of the solar system. Their ice nuclei contain molecules formed at the early stages of planetary formation, and performing spectroscopic observations of the coma, the temporary gaseous atmosphere of a comet, gives insights into the composition of the nucleus. This leads to valuable information about the physical conditions prevailing during planets formation.^1^ In addition, astronomical models show that various volatile species in Earth's atmosphere, especially noble gases, might have originated from comets.^2^ Numerous observations of comets have been performed, covering a wide range of wavelengths from ultraviolet, optical, infrared, to radio.^1^ One of the most significant studies was carried out by the ROSETTA spacecraft, which performed *in situ* observations of comet 67P/Churyumov–Gerasimenko.^3^ These observations led to new insights about the composition of cometary ices and atmospheres, which present a huge diversity of molecules including H_2_O, CO and CO_2_, CH_3_OH and CH_4_.^1,4,5^

Extracting information about the physical conditions and chemical composition of comets, and estimating the abundance of molecules, relies on modeling the observational spectra. The low density conditions in the coma means that the local thermodynamical equilibrium (LTE) is usually not fully achieved, and this modeling requires both radiative and collisional properties of molecules.^6,7^ While radiative data are analytically available for most of the observed molecules, computationally demanding calculations are required to obtain state-to-state collisional rate coefficients. In cometary atmospheres, H_2_O, CO, and CO_2_ are by far the most abundant species, and it is thus crucial to study the mutual collisional excitation of these molecules. In comets, the excitation of molecules is usually dominated by collisions with H_2_O. Several studies involving collisions with water molecules can be found in the literature, from which the collisional systems of H_2_O–H_2_O,^8–10^ and H_2_O–CO,^7^ have the most significant impact for modeling cometary spectra. An important exception is the case of comets at large heliocentric distances, for which the production of gaseous CO is larger than H_2_O. Hence, the excitation of molecules in the coma of such comets is mainly due to collisions with CO.^11–13^ Therefore, it is crucial to investigate the excitation of cometary molecules by CO and as a first priority, the mutual interaction of CO molecules to model the physical conditions in these comets.

Rotational energy transfer in CO–CO collisions has been investigated experimentally in a recent study^14^ that identified unusual pair-correlated excitation mechanisms. Theoretical data for the CO–CO collisional system also exist.^15^ The calculations were performed by combining the time-independent close-coupling method in the low collisional energy regime with the multi-configuration time-dependent Hartree (MCDTH) approach at higher collisional energies. However, the available rate coefficients only cover transitions from the rotational level *j*_1_ = *j*_2_ = 0 to levels with 
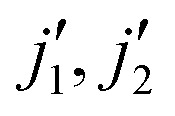
 < 4, *j*_1_ and *j*_2_ being the rotational states of the two colliders. Our goal in the present work is to improve existing data by performing time-independent quantum calculations for rotational energy levels of CO up to, possibly, *j*_1_ = *j*_2_ = 10 that can be used in modeling of cometary atmospheres.

In this paper, we present cross sections and rate coefficients for transitions between the first 37 rotational states of the CO–CO system (*e.g.* states with rotational levels up *j*_1_ and *j*_2_ ≤ 6, for temperatures up to 100 K). In addition, we explore the applicability of a statistical approach to treat such collisional system based on the statistical adiabatic channel method,^16^ to obtain data for rotational levels above *j*_1_ = *j*_2_ = 10. This method was tested and compared with exact quantum calculations and showed excellent agreement especially in low temperatures regime.^16–18^

Our paper is organized as follows: In Section 2, we present the methodology of our work. Section 3 discusses our results using quantum-mechanical and statistical approaches to scattering calculations. In Section 4, we discuss the implications of our work and summarize our results.

## 2 Methodology

### 2.1 Potential energy surface

In addition of the six-dimensional (6D) potential energy surface (PES) of Chen *et al.*,^19^ two accurate 4D CO–CO PESs are available in the literature. The most recent PES^20^ was calculated a few years ago by means of the coupled-cluster approach, and has been tested *versus* experimental spectroscopic studies,^21^ showing its high accuracy. However, it is not suitable for low-energy collisional excitation calculations since the long-range (*R* > 40 *a*_0_) part of the potential is missing. We therefore decided to use the PES calculated by Vissers *et al.*^22^ This PES was calculated at the coupled-cluster single double and perturbative triples [CCSD(T)] level of theory, with the augmented correlation-consistent triple zeta basis (aug-cc-pVTZ). The CO molecules were treated as rigid rotors with the CO bond length fixed at 2.132 *a*_0_. Within the rigid rotor approximation, 4 coordinates (*R*, *θ*_1_, *θ*_2_, *ϕ*) are needed to describe this system. *R* is the length of the vector **R** connecting the centers of mass of the two CO monomers, *θ*_1_ and *θ*_2_ are the polar angles relative to **R**, and the last angle *ϕ* is a dihedral angle between the half-planes that contain both CO molecules. Two minima in the PES were found: the first one, which is a local minimum, at *R* = 6.95 *a*_0_, *θ*_1_ = 59.63°, *θ*_2_ = 120.37° and *ϕ* = 180.0° with a well depth of *V* = −124.21 cm^−1^, and the second one, which is the global minimum of the PES at *R* = 8.20 *a*_0_, *θ*_1_ = −134.23°, *θ*_2_ = 45.77° and *φ* = 180.0° with *V* = −135.53 cm^−1^. The minima are separated by an energetic barrier which is 72.6 cm^−1^ higher than the global minimum. The long range part of the PES has been obtained from an extrapolation of the expansion coefficients assuming a *C*_*n*_/*R*^−*n*^ behavior. The validity of the extrapolation has been verified by checking that the coefficients have physical meaning. The PES of Vissers *et al.*^22^ was also benchmarked against experimental studies^14,21^ that demonstrated its high accuracy.

### 2.2 Scattering calculations

All calculations presented in this work were performed using the MOLSCAT (version 14)^23^ scattering code.

#### 2.2.1 Scattering calculations of two identical molecules

Before proceeding with the details of the scattering calculations, we report an issue with the MOLSCAT code found during this work. Because the scattering system consists in two identical molecules and in order to consider the exchange symmetry of the system, the IDENT option should in principle be applied. Such an option considers the fact that the basis functions corresponding to (*j*_1_, *j*_2_) and (*j*_2_, *j*_1_) are indistinguishable and that only one should be kept, allowing a reduction of the number of channels in the basis by a factor ∼2. Such a reduction allows saving a significant amount of computational time and memory. However, using this option leads to wrong results for some transitions. Indeed, when using the close-coupling (CC)^24^ approach, the cross sections are exactly two times higher for pair–pair transitions (*j*_1_ = *j*_2_ and 
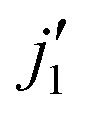
 = 
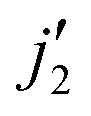
) than what they should be, and when using the coupled states (CS)^25^ approach, the cross sections for all transitions randomly overestimate or underestimate the actual results. This would not be a real issue if the CC calculations were feasible since the results can easily be corrected from the double counting.^26^ However, as will be shown in the following sections, the CC calculations were too CPU consuming and we had to use the approximate CS approach for the calculations.

Hence, in our calculations, we considered both CO molecules as distinguishable molecules, one being the target and one being the projectile. Such an approach is also well suited to astrophysical applications since, in radiative transfer calculations, the notion of colliders and targets is necessarily invoked. Approximate conversion from distinguishable to undistinguishable results will be presented in Section 3.2.

#### 2.2.2 Calculations details

An essential parameter for the calculations is the size of the rotational basis set. We first study the convergence of the cross sections with the size of the rotational basis set using the CC approach. Table 1 presents the results performed at an energy of 20 cm^−1^. The cross sections were summed over total angular momentum values *J* up to 50. As we can observe, a reasonable convergence (better than 2%) is reached with a rotational basis set containing all rotational states up to *j*_1_ = *j*_2_ = 10.

**Table 1:** Cross sections (in Å^2^) at total energy of 20 cm^−1^ for selected *j*_1_,*j*_2_ → 
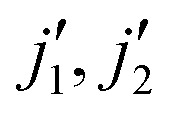
 transitions obtained with a rotational basis that include all levels up to *j*_1_ = *j*_2_ = *j*_max_

Transition	*j*_max_ = 7	*j*_max_ = 8	*j*_max_ = 9	*j*_max_ = 10	*j*_max_ = 11
00 → 11	41.08	35.65	36.21	35.29	35.24
00 → 01	40.06	36.30	30.60	29.24	28.93
01 → 11	85.24	80.16	80.86	80.49	80.23
02 → 01	36.39	34.46	35.32	36.04	35.80

**Fig. 1:**
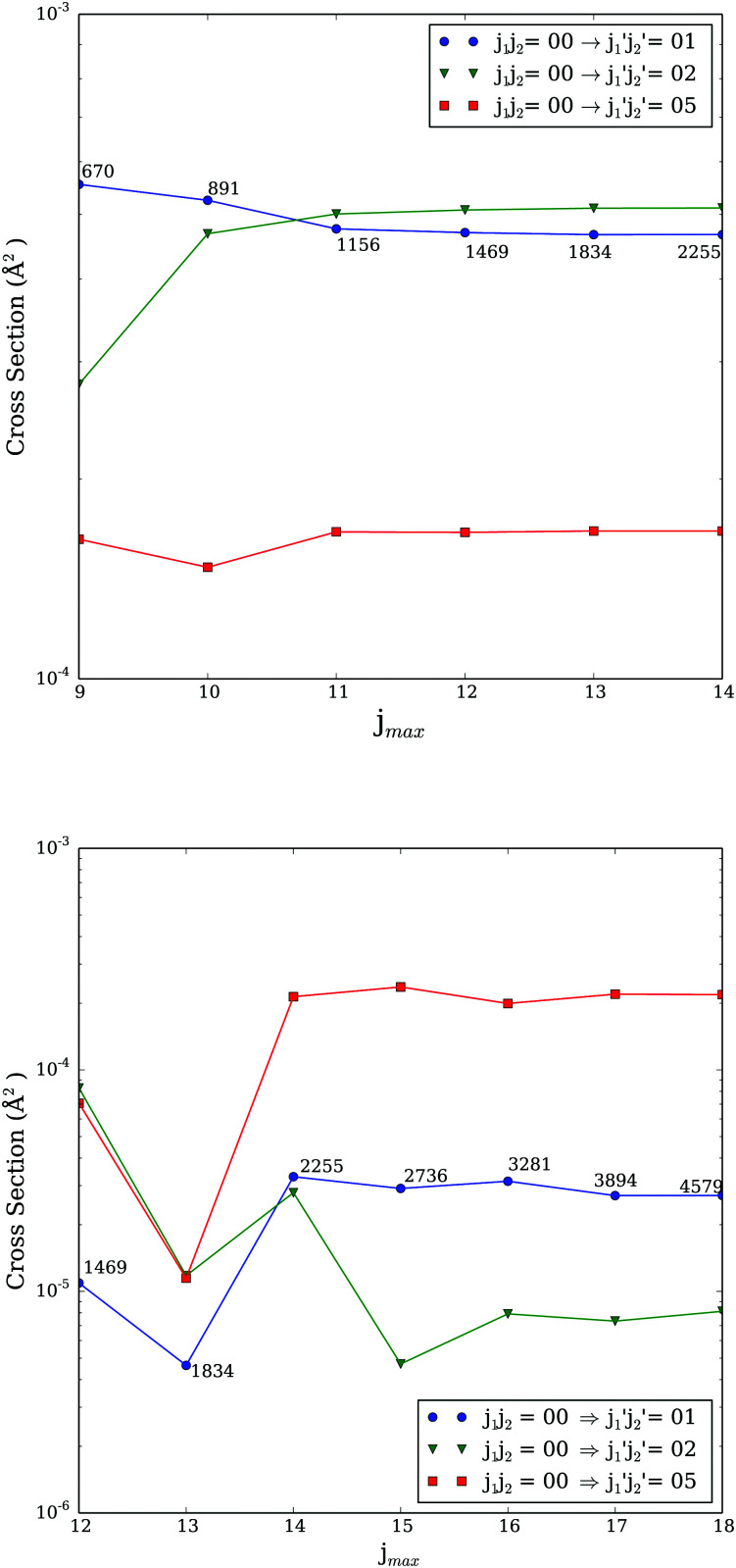
Excitation cross sections for *J* = 0 computed at 100 cm^−1^ (upper panel) and 500 cm^−1^ (lower panel) as function of the rotational basis. The *j*_max_ is equal to highest *j*_1_ and *j*_2_ levels included in the calculations. Numbers on the plot indicate the total number of channels including in the calculations.

Fig. 1 presents additional tests performed at total energies of 100 and 500 cm^−1^. It displays selected cross sections as a function of increasing rotational basis for *J* = 0. As one can see, at 100 cm^−1^, the basis set has to include *j*_1_ = *j*_2_ = 11 for the cross sections to be converged. At 500 cm^−1^, rotational levels up to *j*_1_ = *j*_2_ = 15 have to be included in order to numerically converge calculations. Such a rotational basis leads to 2736 coupled channels‡‡Considering the exchange symmetry, the numbers of channels would still be 1496. and would lead to ∼45 000 coupled channels for *J* ≥ 15. With so many coupled channels, calculations using the (almost) exact CC approach are not feasible for large values of *J* both in terms of memory and CPU time.

**Fig. 2:**
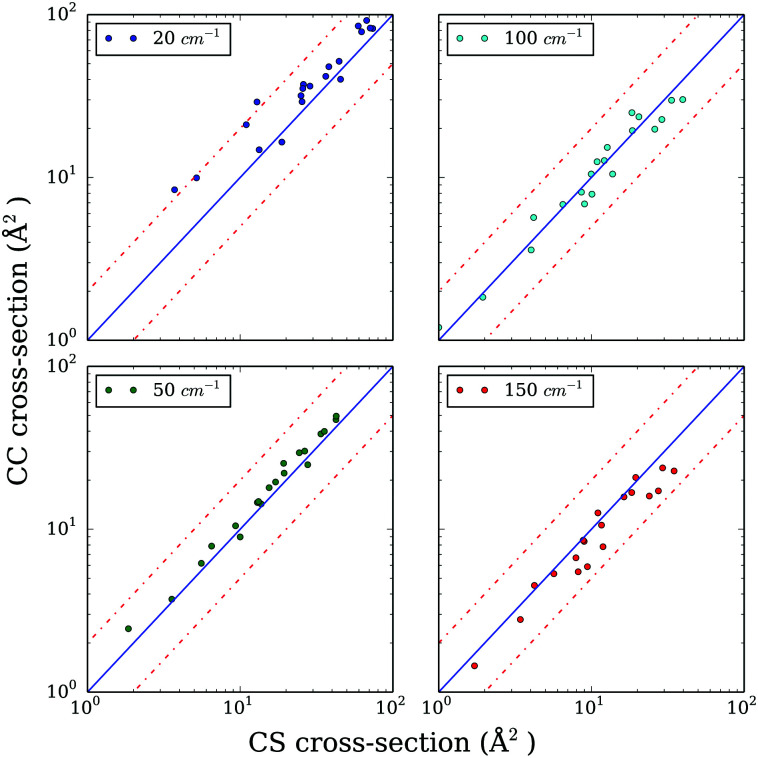
Systematic comparison of selected CS and CC cross sections at 20, 50, 100 and 150 cm^−1^. The dashed lines represent a factor of 2 of difference.

Hence, we explore the possibility of using the CS approximation. In order to evaluate the performance of the CS approach compared to CC, we computed excitation cross sections with a limited basis set containing all rotational levels up to *j*_max_ = 7, expecting that the truncation of the rotational basis would have the same effect on CC and CS results. Fig. 2 presents the CC cross sections as a function of the CS ones for selected values of total energy (20, 50, 100, and 150 cm^−1^). In the low energy regime, where numerous resonances are found, the differences between CC and CS are below a factor of 1.5–2. When the energy increases, as expected, the differences decreases so that the overall agreement is good for energies above 50 cm^−1^. Such comparison indicates that the CS approach is a reasonable alternative for the CC one in the case of CO–CO collisions. For temperatures below 50 K, the estimated accuracy of the rate coefficients obtained from the CS approach is expected to be better than a factor of 2 and this accuracy is expected to increase with increasing temperature.

The coupled equations were then solved using the CS approximation with the log-derivative Airy propagator.^27^ The STEPS parameter of MOLSCAT was set at 20 in order to obtain a step length of the integrator sufficient to achieve the convergence. The integration was performed for distances between *R*_min_ = 5 *a*_0_ and *R*_max_ = 50 *a*_0_. The rotational constants of the CO molecules were taken as: *B*_e_ = 1.931 cm^−1^, *α*_e_ = 1.750 × 10^−2^ cm^−1^, *D*_e_ = 6.121 × 10^−6^ cm^−1^.^28^ The reduced mass was set at *μ* = 13.997 u. Excitation cross sections were obtained between rotational states listed in Table 2. The following energy grid was used: in 0–200 cm^−1^ energy range, we used steps of 1 cm^−1^, in the 200–300 cm^−1^ energy range, the energy step was increased to 5 cm^−1^, and finally in the 300–800 cm^−1^ energy range, a step of 100 cm^−1^ was used. The number of total angular momentum *J* needed to converge calculations vary from 35, at low energy, to 130, at the highest energies. From the computed cross sections, we obtain rate coefficients for temperatures up to 100 K using the following the formula:1
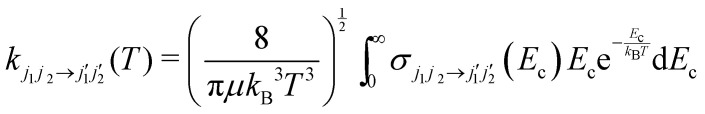
where, *μ* is reduced mass of the system, *k*_B_ is the Boltzmann constant, 
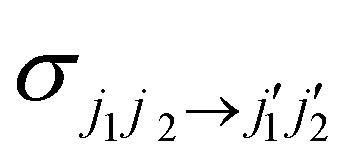
 is the cross section and *E*_c_ is collision energy.

**Table 2:** Dissociating rotational states of the CO(*j*_1_)–CO(*j*_2_) system

Level	*j*_1_ *j*_2_	Energy (cm^−1^)	Level	*j*_1_ *j*_2_	Energy (cm^−1^)
1	00	0.000	20	35	80.739
2	01	3.845	21	16	84.580
3	11	7.690	22	26	92.270
4	02	11.535	23	45	96.118
5	12	15.379	24	36	103.805
6	03	23.069	25	07	107.642
7	22	23.070	26	17	111.487
8	13	26.914	27	55	115.341
9	23	34.604	28	27	119.177
10	04	38.448	29	46	119.186
11	14	42.293	30	37	130.712
12	33	46.139	31	08	138.390
13	24	49.983	32	56	138.406
14	05	57.670	33	18	142.235
15	15	61.515	34	47	146.091
16	34	61.517	35	28	149.925
17	25	69.205	36	38	161.460
18	44	76.896	37	66	161.471
19	06	80.735			

## 3 Results and discussion

Using the methodology described above, we computed state-to-state excitation cross sections for CO–CO collisions. The calculations were performed for energies up to 800 cm^−1^ and cover transitions between levels up to *j*_1_ = *j*_2_ = 6. Collisional rate coefficients up to 100 K were derived from these cross sections. Collisional rate coefficients are available as ESI.† All the results presented below have been obtained considering the two CO molecules as distinguishable.

### 3.1 Propensity rules

**Fig. 3:**
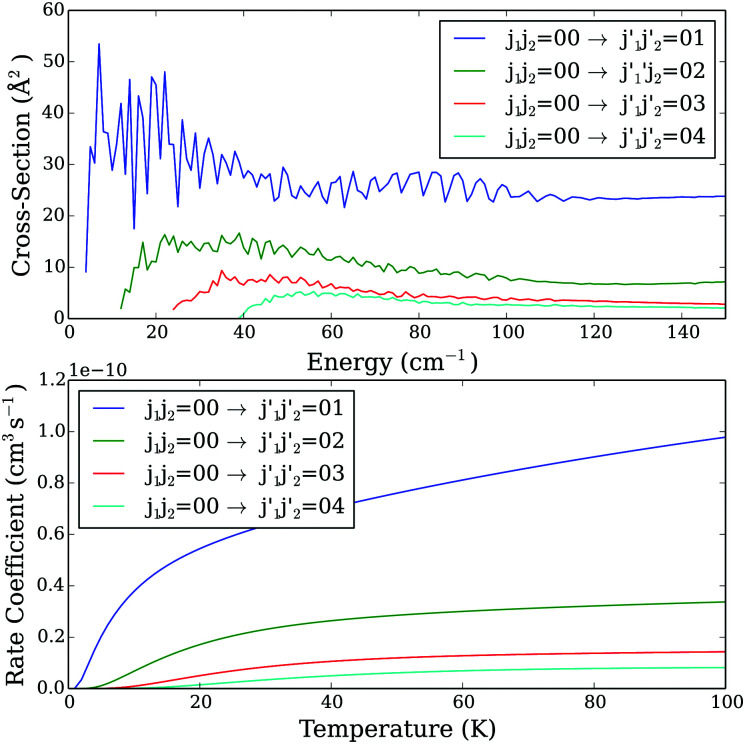
Excitation cross sections as a function of collision energy (upper panel) and the rate coefficients as a function of temperature (lower panel) from the *j*_1_*j*_2_ = 00 state.

**Fig. 4:**
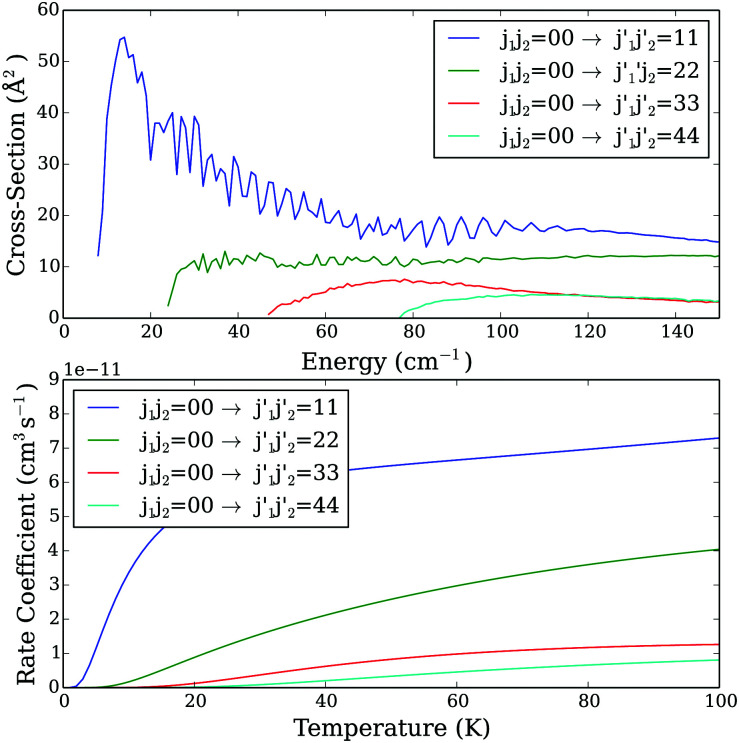
Excitation cross sections as a function of collision energy (upper panel) and the rate coefficients as a function of temperature (lower panel) from the *j*_1_*j*_2_ = 00 state.

In Fig. 3, we present selected CO–CO cross sections corresponding to the excitation from the ground state *j*_1_*j*_2_ = 00 to rotational states in which only one CO molecule is excited. As one can see, the dominant transition is the transition to the 
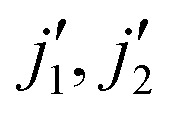
 = 01 state. The values of the cross sections decrease when 
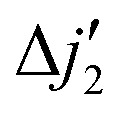
 increases, in agreement with an exponential energy-gap behavior. Obviously, the same propensity rules can be seen when we compare rate coefficients (Fig. 3, lower panel). A similar propensity rule can also be seen when both CO molecules are excited after the collision. Indeed, as one can see on Fig. 4 that presents selected excitation cross sections and rate coefficients from the fundamental *j*_1_*j*_2_ = 00 state, the dominant transition is the one corresponding to the excitation to the 
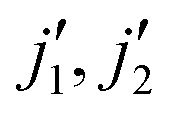
 = 11 state. More generally, the cross sections decrease with the increase of both 
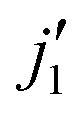
 and 
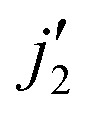
 final levels.

**Fig. 5:**
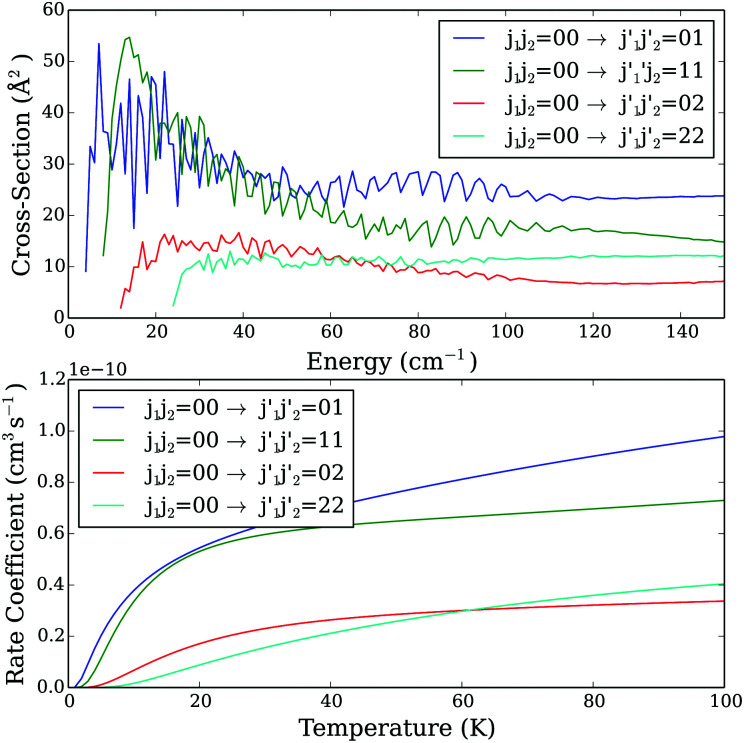
Excitation cross sections as a function of collision energy (upper panel) and rate coefficients as a function of temperature (lower panel) from level *j*_1_*j*_2_ = 00. The presented transitions show comparisons of transitions where one of the CO molecules is excited with transitions where both of them are excited.

In Fig. 5, we compare the cross sections and rate coefficients for transitions where only one CO molecule is excited to the ones for transitions where both CO molecules are excited. In contrast to previous findings reported by Ndengué *et al.*,^15^ we do not observe propensity rules in favor of pair–pair transitions (*j*_1_ = *j*_2_ and 
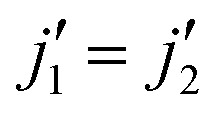
). We found that the magnitude of the cross sections involving excitation of only one CO molecule is close to the one of the cross sections where both CO molecules are excited.

Propensity rules similar to those reported in our work were observed in previous studies of collisions of two identical molecules. For example, in H_2_–H_2_ scattering, the cross sections were found to be larger for transitions where only one molecule is excited compared to the transitions where both colliders are excited.^29^

### 3.2 Comparison with previous data

As stated in the introduction, theoretical data for the CO–CO collisional system have been published by Ndengué *et al.*^15^ It is then of interest to compare our new data with these existing ones. Ndengué *et al.*^15^ performed scattering calculations using the PES of Dawes *et al.*^20^ The authors combined time-independent quantum CC calculations for energy up to 150 cm^−1^, with MCTDH calculations for energies above 150 cm^−1^. In the CC calculations, they included in the rotational basis set CO levels up to *j*_1_ = *j*_2_ = 7. Using the MCTDH method, the basis was substantially increased due to the lower computational cost. The calculations of Ndengué *et al.*^15^ were performed treating the CO molecules as undistinguishable particles. We do not focus here on the differences between collisional cross sections above 150 cm^−1^ that are most probably due to different scattering methods (quantum time dependent *vs.* quantum time independent) and that weakly affect the collisional rate coefficients below 100 K.

**Fig. 6:**
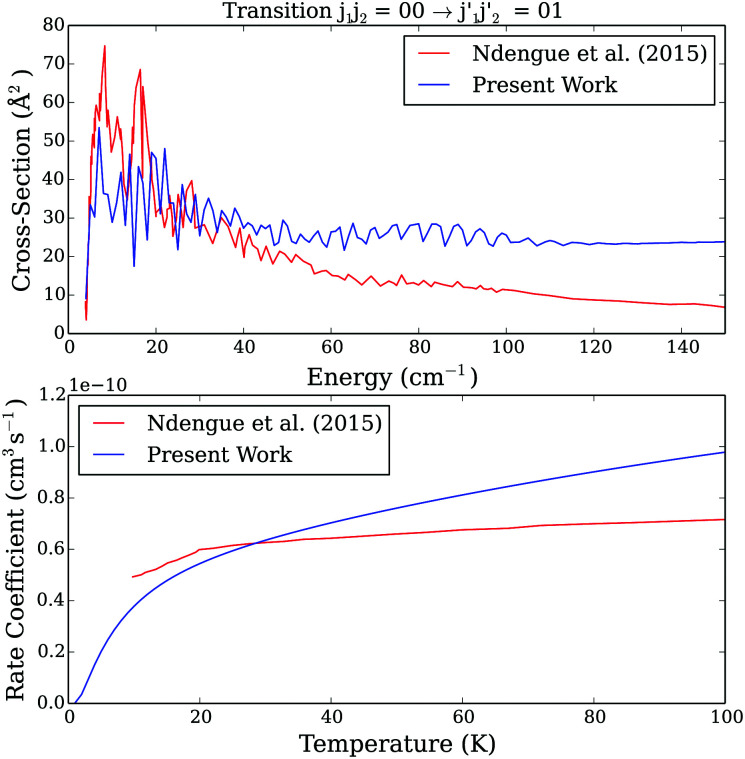
Comparison between present excitation cross sections as a function of energy and those of Ndengué *et al.*^15^ (upper panel) and rate coefficients as a function of temperature (lower panel) for the *j*_1_*j*_2_ = 00 to 
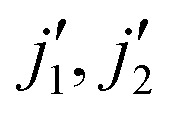
 = 01 transition.

**Fig. 7:**
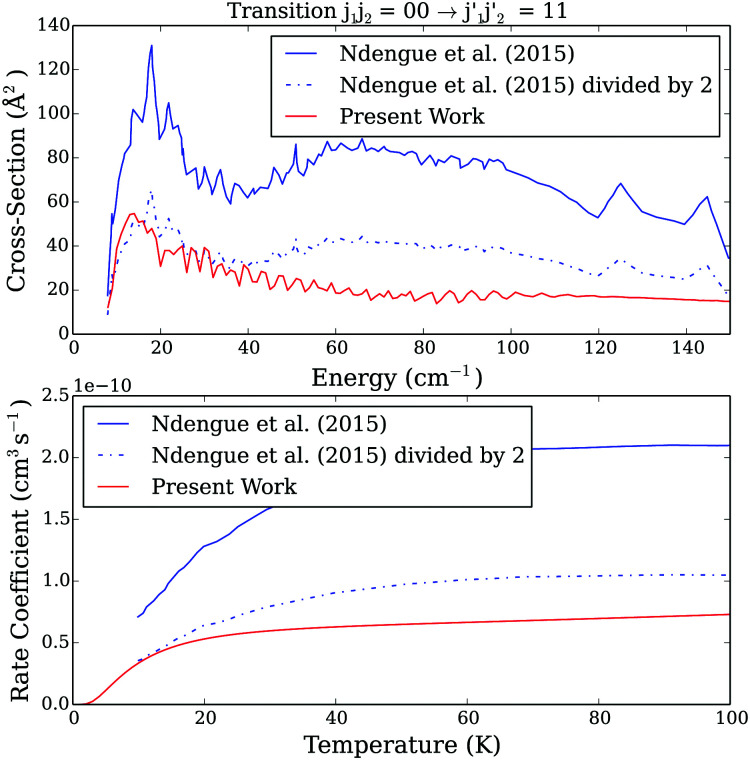
Comparison between present and Ndengué *et al.*^15^ excitation cross sections as a function of energy (upper panel) and rate coefficients as a function of temperature (lower panel) for the *j*_1_*j*_2_ = 00 to 
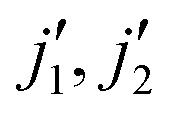
 = 11 transition. The solid blue line represents results directly taken from Ndengué *et al.*,^15^ the dashed blue line represents the same results divided by a factor of 2, and the red solid line represents our results.

Fig. 6 and 7 present a comparison of our new collisional data (both the cross sections and rate coefficients) to the ones of Ndengué *et al.*^15^ for two selected transitions. Since Ndengué *et al.*^15^ considered scattering between undistinguishable particles and performed the calculations with the MOLSCAT code, we also plotted their results for pair–pair transitions divided by two (*e.g.* discussion in Section 2.2.1). For the purpose of the comparison with Ndengué *et al.*^15^ results, we also converted our distinguishable results to undistinguishable ones using the following formulae, that are not strictly exact and based on the assumption that quantum interference effects are negligible^30,31^ §§Note that there is not a clear convergence of the conversion factor to be used (*e.g.* discussion in Perez-Rios *et al.*^31^) and we do not recommend to use our estimated undistinguishable observables for experiments analysis.

• Pair–pair transitions (*j*_1_ = *j*_2_ and 
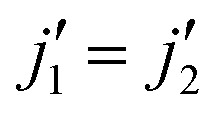
)2*τ*_u_(*j*_1_*j*_2_ → 
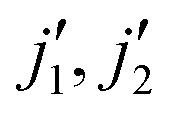
) = *τ*_d_(*j*_1_*j*_2_ → 
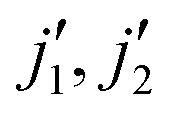
)

• Pair–no-pair transitions (*j*_1_ = *j*_2_ and 
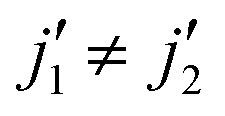
)3*τ*_u_(*j*_1_*j*_2_ → 
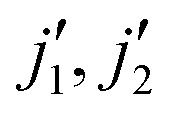
) = *τ*_d_(*j*_1_*j*_2_ → 
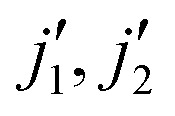
) + *τ*_d_(*j*_1_*j*_2_ → 
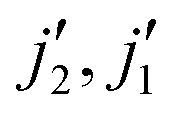
)

• No-pair–pair transitions (*j*_1_ ≠ *j*_2_ and 
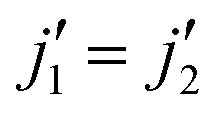
)4*τ*_u_(*j*_1_*j*_2_ → 
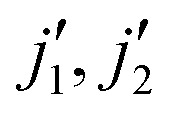
) = *τ*_d_(*j*_1_*j*_2_ → 
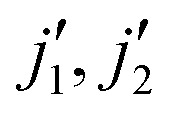
)

• No-pair–no-pair transitions (*j*_1_ ≠ *j*_2_ and 
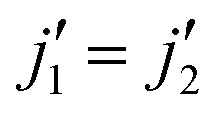
)5*τ*_u_(*j*_1_*j*_2_ → 
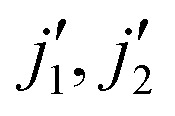
) = *τ*_d_(*j*_1_*j*_2_ → 
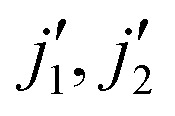
) + *τ*_d_(*j*_1_*j*_2_ → 
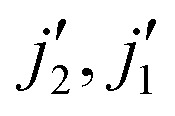
)where *τ*_d_ and *τ*_u_ are the distinguishable and undistinguishable observables (cross sections or rate coefficients).

As one can see, differences between the two sets of data are significant (even when corrected for the possible IDENT MOLSCAT parameter issue). Such differences originate from the different approaches (different CO–CO PESs, different scattering theory and different calculations parameters) used in the scattering studies. It is then of interest to assess the importance of all these different computational aspects in the overall discrepancy between the two sets of data.

The differences induced from the use of different PESs is found to be minor. Indeed, we performed test calculations using the Vissers *et al.*^22^ PES and calculations parameters reported by Ndengué *et al.*^15^ (for energies up to 50 cm^−1^) and we found that the differences were lower than 10% on the average despite the fact that the resonances seen in the excitation cross sections were slightly shifted. We expect that the difference would be even lower in the case of rate coefficients, where the cross sections are averaged over a thermal distribution of collisional energies.

The impact of the use of the CS *vs.* CC scattering approach was evaluated in the Section 2. Indeed, Fig. 2 showed that the CC and CS results can differ by up to a factor of 1.5–2 at low collision energies as can be observed when comparing our results to the results from Ndengué *et al.*^15^ (Fig. 6 and 7). However, it was also found that the agreement between CC and CS results increases with increasing collision energies. Such an improvement is not seen here.

This can probably be explained this by the size of the rotational basis set used in the two quantum time independent calculations. Ndengué *et al.*^15^ used a rotational basis containing all levels with *j*_1_ and *j*_2_ up to 7 that clearly does not allow full convergence of the collisional cross sections (*e.g.*Table 1), the non convergence of the results obviously increasing with increasing energies. Indeed, in order to fully converge our scattering calculations, we used basis *j*_1_ = *j*_2_ = 15 and a significant part of the difference between the two sets of data above 100 cm^−1^ can be explained by the lack of convergence of the Ndengué *et al.*^15^ calculations with respect to the rotational basis.

### 3.3 Statistical approach

As we reported, the accuracy of our data for temperatures below 50 K is within a factor of 1.5–2. The pure CC calculations being not feasible, alternative approaches were therefore explored in order to improve accuracy. We decided to investigate the validity of statistical methods to check whether the accuracy of our rate coefficients could be improved in the low temperatures regime and whether the calculations could be extended to transitions involving higher rotational levels. We used the Statistical Adiabatic Chanel Model (SACM) presented in the work of Loreau *et al.*^16,32^ The significant advantage of this method is that the calculations are performed only for one value of the energy since the adiabatic curves are energy-independent. In addition, we could use a much smaller rotational basis set to reach convergence of the cross sections (*j*_max_ = 6 was enough to obtain converged calculations for transitions between levels up to *j*_1_ = *j*_2_ = 5).

**Fig. 8:**
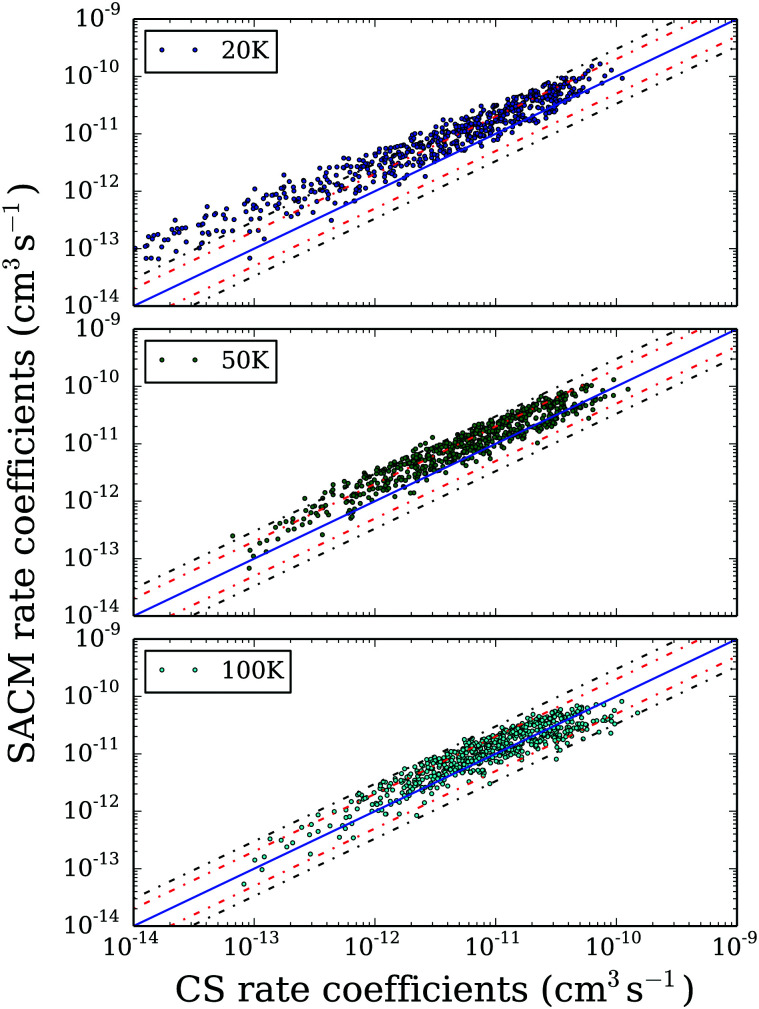
Systematic comparison of SACM and CS rate coefficients, at temperatures of 20, 50, and 100 K, for all transitions calculated in this work (using the CS method). The blue line represents exact results, red dashed lines represent a factor of 2 difference, and black dashed lines represent a factor of 3 difference.

We calculated cross sections for transitions between rotational levels up to *j*_1_ = *j*_2_ = 5, and rate coefficients for temperatures up to 100 K. In Fig. 8, we show a systematic comparison between rate coefficients obtained using SACM and CS methods. Previous use of the SACM method showed that it works well at low temperatures and starts to deviate from accurate results when the temperature increases.^16^ However, in our case, the opposite behavior is observed. As one can see from Fig. 8, at 20 K, the differences between the two sets of data are above a factor of 3 for numerous transitions while the agreement is increasing with increasing temperature.

**Fig. 9:**
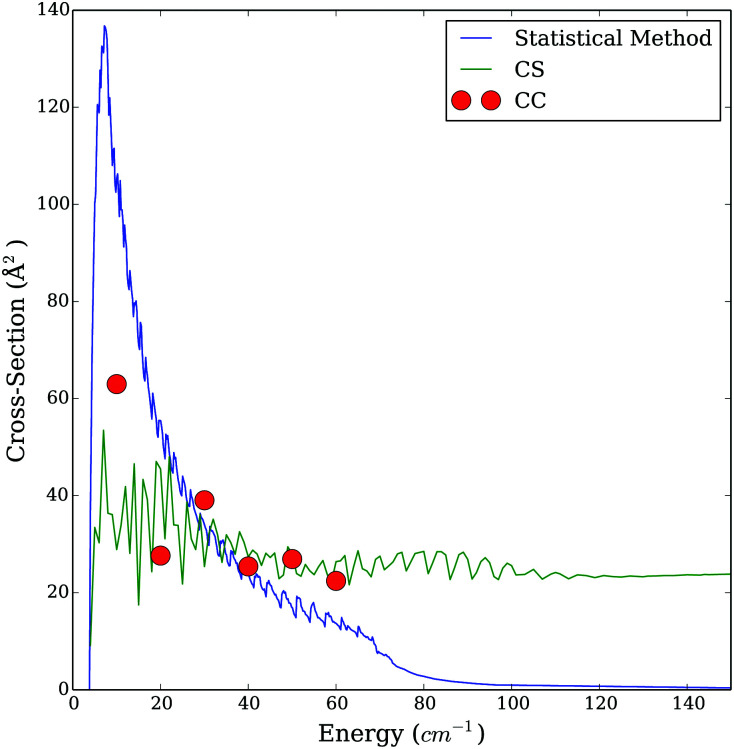
Comparison of the cross section obtained with the CC, CS and statistical method for the transition *j*_1_*j*_2_ = 00 to 
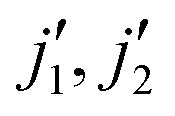
 = 01 as a function of energy.

**Fig. 10:**
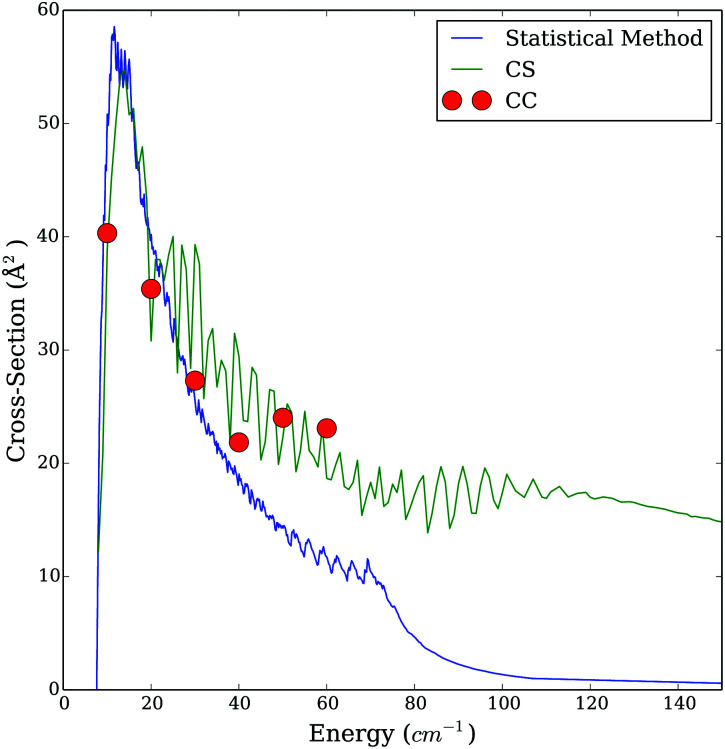
Comparison of the cross section obtained with the CC, CS and statistical method for the transition *j*_1_*j*_2_ = 00 to 
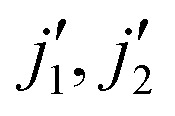
 = 11 as a function of energy.

We have to keep in mind that we compare here two approximate methods. Therefore, we calculated cross sections for a few energies using the almost exact CC method including in the rotational basis levels up to *j*_1_ = *j*_2_ ≤ 10. Fig. 9 and 10 show an example of comparison of the CS, SACM and the CC cross sections. Generally, we observed that the results obtained with the CS method underestimate the cross sections, while the SACM results slightly overestimate the cross sections compared to the CC ones in the low energy regime. However, both the CS and SACM results stays within a factor of 2 compared to the CC results; therefore, we cannot clearly determine which method is more accurate. In the intermediate region of energy (30 ≤ *E*_c_ ≤ 70 cm^−1^), all three methods agrees reasonably well, thereby explaining the fairly good agreement seen in Fig. 8 for temperatures of 50 and 100 K. Important deviations start to occur at higher energies. Indeed, we observe a substantial decrease in the cross sections obtained with the SACM method, while the CC and CS results stay in good agreement. We presume that this decrease of cross sections is due to the dense distribution of the accessible (open) channels and also to the fact that the well depth of the CO–CO PES is not large enough for statistical approaches to work above the very low energy regime.^16^

We conclude that the rate coefficients obtained with the SACM method may be useful for temperatures up to 100 K. The good agreement between SACM and CS rate coefficients observed at a temperature of 100 K is rather fortuitous and difference above 100 K can become very significant because of the strong deviation between quantum and SACM results at high collisional energies. Therefore, we are not able to extend the rate coefficients to higher temperatures and hence higher rotational levels (populated only at high temperature) using the SACM method.

## 4 Conclusions

Using the potential of Vissers *et al.*,^22^ we presented the first complete quantum scattering calculations of CO–CO rotational excitation between first 37 rotational energy states of CO molecules. We obtained state-to-state collisional rate coefficients for temperatures up to 100 K. We estimated that our data below 50 K are accurate within a factor of 1.5–2 and that the accuracy increases with increasing temperature.

Significant differences were found between our results and those previously reported by Ndengué *et al.*^15^ Transitions towards “pair” rotational levels (with *j*_1_ = *j*_2_) seem to be overestimated by a factor 2. For temperatures above 50 K, we believe that our results are more accurate than the previous ones. Our convergence tests show that the basis used in the previous study was insufficient. At the same time, we are confident that the basis size used in our work allows us to converge calculations over the whole energy regime considered. The question about the accuracy of the rate coefficients below 50 K remain. Even though the previous calculations were performed using the CC method, their results were only partially converged due to the small basis set. On the contrary, our calculations were converged. However, we used the CS approximation, which was estimated to provide accurate results within a factor of 2 difference.

We tried to improve our data by investigating the possibility of the statistical approach to the CO–CO scattering. The results we obtained do not lead to a clear answer. The agreement was excellent for part of the transitions in the low energy regime. For other transitions, differences of a factor of ∼2 were observed between SACM and partly converged CC calculations. In addition, for all the considered transitions, we observed a sudden decrease of cross section above 60–70 cm^−1^. This behavior can be expected when the collision energy becomes comparable to the well depth of the PES, but further investigation on similar systems would be needed.

The CS calculations will be continued using the methodology reported in this work. We will extend data for transitions between levels up to *j*_1_ = *j*_2_ = 10. The impact of our data on the astrophysical models, in particular of cometary atmospheres, will be presented in a separate article.

## Data availability

The data that support the findings of this study are available within the article and its online ESI.†

## Conflicts of interest

There are no conflicts to declare.

## Supplementary Material

CP-024-D2CP01065H-s001
